# Editorial: Advances in Vaccine Delivery: Adjuvants, Carriers, Formulations, and Routes

**DOI:** 10.3389/fphar.2022.857792

**Published:** 2022-02-09

**Authors:** Mariusz Skwarczynski

**Affiliations:** School of Chemistry and Molecular Biosciences, The University of Queensland, St. Lucia, QLD, Australia

**Keywords:** vaccine, adjuvant, carrier, nanomedcine, delivery

Vaccines are not only the most powerful tool for combatting infectious diseases, they are also used to fight cancer and control fertility in animals. Traditional whole microorganism-based vaccines have been highly effective; however, they are not always entirely safe and may induce undesirable immune responses and side effects. More recent alternative strategies include subunit (protein, peptide, and carbohydrate-based antigens) and genetic vaccines (DNA and RNA-based), which only contain or encode the antigens required to stimulate the desired effects. However, pathogen-derived antigens, or their fragments, are poorly immunogenic and need to be administered with immune stimulants (adjuvants), which often contain fragments of microorganisms (Reed et al., 2013). Moreover, the application of minimal antigen in vaccine design often requires the use of a protein carrier, which stimulates stronger immunity against the carried antigen (Pichichero, 2013). These carriers usually act as a source of T-helper epitopes, enhancing the quality and longevity of antigen-specific immune responses, while ideally not generating strong immune responses against themselves.

Adjuvants are typically composed of individual or mixed lipids, poly/liposaccharides, or various microbial components. Adjuvants usually mimic natural danger signals, known as pathogen-associated molecular patterns (PAMPs), which are recognized by the immune system. Unfortunately, the safest adjuvants are often inadequate or possess serious limitations. This includes poor induction of cellular immunity or ineffectiveness when delivered through oral or intranasal routes. In contrast, many potent adjuvants are toxic, non-biodegradable, and consistently invoke adverse reactions: mostly strong allergic and inflammatory responses. Consequently, alum compounds were the only adjuvants approved for human use for many decades. Fortunately, recent advances in medicinal chemistry, immunology, pharmacology, and nanotechnology have supported the development of a variety of new immune stimulants/adjuvants, delivery systems, and formulations for subunit vaccines (Sun and Xia, 2016; Nevagi et al., 2019; Wang and Xu, 2020). New adjuvants and their formulations have recently been introduced to clinically approved vaccines; however, these have only been approved for particular formulations/vaccines, and often only in certain countries. Thus, an urgent need remains for non-toxic, biodegradable and biocompatible adjuvants or self-adjuvanting delivery systems that can help stimulate effective, long-lasting, and safe immunity.

Liposaccharides are one of the most extensively investigated groups of nature-derived adjuvants. Liu et al. identified *Alcaligenes*-derived lipopolysaccharide, isolated from bacterium residing in the lymphoid tissues, such as Peyer’s patches in mucosal membranes. The capacity of this lipopolysaccharide to stimulate humoral immunity through activation of the toll receptor pathway was proven for *Haemophilus influenzae* B conjugate vaccine built from capsular polysaccharide polyribosyl ribitol phosphate conjugated to carrier tetanus toxoid.

Vaccine delivery pathways beyond the standard intramuscular route are also being increasingly investigated (Skwarczynski and Toth, 2020). Vaccine composition strongly depends on immunization route; thus, intensive effort is going into the development of vaccines that can be administered orally, intranasally, intradermally, etc., to avoid the disadvantages of injectable vaccines (Giudice and Campbell, 2006; Vela Ramirez et al., 2017; Yusuf and Kett, 2017).

Oral and intranasal delivery can be especially advantageous, as these routes mimic natural infection paths, are more patient friendly, induce mucosal immunity (preventing pathogen entry into the host), and sterility is less critical. Mucosal vaccines can also be cheaper, and even self-administered, providing greater feasibility for mass immunizations. Still, enzymatic degradation of the vaccine, rapid clearance form mucosal surfaces, limited permeability, and lack of approved mucosal adjuvants are all serious limitations that need to be overcome in order to produce effective mucosal vaccines.

The formulation of vaccines into nano- and microparticles is an alternative strategy to the above-mentioned nature-derived adjuvant approach. Jazayeri et al. reviewed recent progress in the use of particles for oral vaccine delivery. As the mucosal surfaces are the first line of defense against invading pathogens, producing mucosal immune responses, especially after oral vaccine delivery, provides a variety of advantages. However, oral vaccines need to pass extremely low pH environments, proteolytic enzymes, bile salts, and overcome low permeability, immunogenicity and oral tolerance. The review briefly discusses the main mechanisms of mucosal immunity, then provides substantial detail on a variety of nano/microparticle-based approaches to overcome the above-mentioned obstacles.

Along similar lines, Abisoye-Ogunniyan et al. reviewed recent approaches in the delivery of vaccines against sexually transmitted infections. The review starts with a general overview of the most common nanocarriers used in vaccine delivery, then focuses in on vaccines against sexually transmitted infections. Finally, the potential role of nano delivery systems in vaccine development against sexually transmitted infections is discussed.

Howlader et al. demonstrate a real-life application of nanoparticle-based delivery systems. They applied two nanoformulations to their previously developed L-PaF vaccine against *Pseudomonas aeruginosa* composed of a mixture of protein antigens. The first antigen formulation was based on an oil-in-water (o/w) emulsion and the second on a chitosan particle: both were adjuvanted with TLR4 agonist, BECC438 (a detoxified lipid A). The emulsion-based L-PaF/BECC438 formulation (∼100 nm in diameter) was highly immunogenic and provided the best protective efficacy in mice.

Systemic and oral immunity of a vaccine against porcine epidemic diarrhea virus (PEDV) was investigated by Tien et al. The vaccine was designed based on molecularly engineered adjuvant, PIGS (polymeric immunoglobulin scaffold), and epitope antigen from the S1 protein of PEDV, while bacterial cholera toxin (bCT) was used as an additional adjuvant. It induced humoral immune responses following both systemic and mucosal (oral) administration. Mucosal vaccine administration was also investigated by Gomez et al. However, instead of the classical solution-based formulation, a dry powder-based system was used to deliver ID93+GLA-SE, a promising tuberculosis vaccine candidate. Intranasal and pulmonary delivery routes were tested for spray-dried powder and the formulations were individually optimized for each route. The protective efficacy of the vaccine formulations following delivery by both routes was comparable to that of vaccine administered through intramuscular delivery.

## References

[B1] Giudice E. L. Campbell J. D. (2006). Needle-free Vaccine Delivery. Adv. Drug Deliv. Rev. 58, 68–89. 10.1016/j.addr.2005.12.003 16564111

[B2] Nevagi R. J. Skwarczynski M. Toth I. (2019). Polymers for Subunit Vaccine Delivery. Eur. Polym. J. 114, 397–410. 10.1016/j.eurpolymj.2019.03.009

[B3] Pichichero M. E. (2013). Protein Carriers of Conjugate Vaccines: Characteristics, Development and Clinical Trials. vaccines 9, 2505–2523. 10.4161/hv.26109 PMC4162048 23955057

[B4] Reed S. G. Orr M. T. Fox C. B. (2013). Key Roles of Adjuvants in Modern Vaccines. Nat. Med. 19, 1597–1608. 10.1038/nm.3409 24309663

[B5] Skwarczynski M. Toth I. (2020). Non-invasive Mucosal Vaccine Delivery: Advantages, Challenges and the Future. Expert Opin. Drug Deliv. 17, 435–437. 10.1080/17425247.2020.1731468 32059625

[B6] Sun B. Xia T. (2016). Nanomaterial-based Vaccine Adjuvants. J. Mater. Chem. B 4, 5496–5509. 10.1039/C6TB01131D 30774955 PMC6377210

[B7] Vela Ramirez J. E. Sharpe L. A. Peppas N. A. (2017). Current State and Challenges in Developing Oral Vaccines. Adv. Drug Deliv. Rev. 114, 116–131. 10.1016/j.addr.2017.04.008 28438674 PMC6132247

[B8] Wang Z. B. Xu J. (2020). Better Adjuvants for Better Vaccines: Progress in Adjuvant Delivery Systems, Modifications, and Adjuvant-Antigen Codelivery. Vaccines 8 (1), 128. 10.3390/vaccines8010128 PMC7157724 32183209

[B9] Yusuf H. Kett V. (2017). Current Prospects and Future Challenges for Nasal Vaccine Delivery. Hum. Vaccin. Immunother. 13, 34–45. 10.1080/21645515.2016.1239668 27936348 PMC5287317

